# Genetic Evaluation of Growth Traits in Black-Boned and Thai Native Synthetic Chickens Under Heat Stress

**DOI:** 10.3390/ani15152314

**Published:** 2025-08-07

**Authors:** Wootichai Kenchaiwong, Doungnapa Promket, Vatsana Sirisan, Vibuntita Chankitisakul, Srinuan Kananit, Wuttigrai Boonkum

**Affiliations:** 1 Small Ruminant Research Unit, Faculty of Veterinary Science, Mahasarakham University, Mahasarakham 44000, Thailand; wootken@gmail.com (W.K.); siri.vatsana@gmail.com (V.S.); 2 Network Center for Animal Breeding and Omics Research, Khon Kaen University, Khon Kaen 40002, Thailand; vibuch@kku.ac.th (V.C.); srinka@kku.ac.th (S.K.); 3 Applied Animal and Aquatic Sciences Research Unit, Faculty of Technology, Mahasarakham University, Kantarawichai, Mahasarakham 44150, Thailand; napakran@hotmail.com; 4 Department of Animal Science, Faculty of Agriculture, Khon Kaen University, Khon Kaen 40002, Thailand

**Keywords:** absolute growth rate, average daily gain, body weight, heat tolerance, synthetic chicken line

## Abstract

Heat stress is a critical constraint to poultry production in tropical regions, where the temperature–humidity index (THI) frequently exceeds thermoneutral thresholds. Despite growing interest in climate-resilient livestock, limited research has explored the genetic sensitivity of local chicken breeds to increasing THI levels. This study aimed to evaluate the genetic effects of increasing THI on growth performance traits in two tropical chicken breeds. The data included body weight (BW), average daily gain (ADG), and absolute growth rate (AGR) from 4,745 black-boned and 3,001 Thai native synthetic chickens across five generations. Growth data were collected from hatching to 12 weeks of age, whereas temperature and humidity were continuously recorded to calculate daily THI values. A reaction norm model was used to estimate genetic parameters and rate of decline of BW, ADG, and AGR traits under varying THI thresholds (THI70 to THI80). Results indicated that the onset of heat stress occurred at THI72 for black-boned chickens and at THI76 for Thai native synthetic chickens. Heritability estimates for BW, ADG, and AGR decreased as the THI increased in both chicken breeds. However, the Thai native synthetic chickens consistently exhibited higher genetic potential across all THI levels (average heritability: BW = 0.28, ADG = 0.25, AGR = 0.36) compared to the black-boned chickens (average heritability: BW = 0.21, ADG = 0.15, AGR = 0.23). Under mild heat stress (THI72), black-boned chickens showed sharp declines in all traits (average reduction in BW = −10.9 g, ADG = −0.87 g/day, AGR = −3.20 g/week), whereas Thai native synthetic chickens maintained stable performance. At THI76, both breeds experienced significant reductions, particularly in males. Estimated breeding values (EBVs) for AGR decreased linearly with THI, though Thai native synthetic chickens showed greater individual variability, with some birds maintaining stable or positive EBVs up to THI80—suggesting the presence of heat-resilient genotypes. In conclusion, Thai native synthetic chickens demonstrated superior thermotolerance and genetic robustness under increasing THI conditions. The identification of breed-specific THI thresholds and resilient individuals provides novel insights for climate-smart poultry breeding. These findings offer valuable tools for genetic selection, environmental management, and long-term adaptation strategies in response to global climate change.

## Simple Summary

Rising temperatures and humidity due to climate change pose significant challenges to poultry farming, particularly in tropical regions. This study evaluated and compared the growth performance and heat tolerance of two chicken types—black-boned and Thai native synthetic chickens. Over several years, researchers monitored their growth under varying environmental conditions. The findings revealed that Thai native synthetic chickens exhibited superior growth rates and greater resilience to heat stress compared to black-boned chickens. In contrast, black-boned chickens showed reduced growth even under mild heat conditions, whereas the synthetic chickens maintained stable performance until exposed to more severe heat. These insights can guide farmers in selecting more heat-tolerant chicken breeds, thereby improving food production efficiency, minimizing losses, and promoting sustainable poultry farming in the face of global warming.

## 1. Introduction

Poultry production plays a critical role in ensuring global food security and providing economic opportunities for smallholder farmers, particularly in developing countries [1,2,3,4]. In Thailand and other tropical regions, black-boned and synthetic chickens have gained increasing attention owing to their adaptability, unique meat quality, and potential for improving local poultry genetic resources [5,6,7,8]. These breeds not only contribute to household income but also support the development of sustainable and resilient poultry production systems, aligning with the principles of a circular economy at the community level [1]. Growth performance is an important economic trait in poultry breeding programs because it directly impacts meat yield, feed efficiency, and overall productivity [9,10]. Genetic selection for optimal growth performance in black-boned and synthetic chickens can enhance profitability for farmers by reducing production costs and increasing market value. This knowledge is applicable not only to Thai farmers but also to farmers in other countries to improve their native chicken production systems [5,6]. Moreover, improving growth traits in these breeds aligns with the broader goal of genetic sustainability, ensuring long-term adaptability and productivity [11,12].

Poultry production in tropical regions faces significant challenges from heat stress, which impairs growth performance, feed efficiency, and bird welfare [13,14,15]. As global temperatures and humidity levels continue to rise, heat tolerance must be prioritized in genetic improvement strategies. Developing integrated genetic models that incorporate both growth traits and thermotolerance is essential to sustain productivity under climate change conditions [4,10,16,17]. Among environmental stressors, elevated temperature–humidity index (THI) levels significantly impair growth performance, reduce feed intake, and influence the genetic expression of heat tolerance in chickens [18,19]. Previous research has extensively investigated the physiological and nutritional effects of heat stress on poultry growth performance [20,21]. Elevated temperature and humidity impair thermoregulation, increasing panting, oxidative stress, and body temperature, which in turn reduce feed intake and hormonal balance, ultimately lowering metabolic efficiency and growth [13,22]. Nutritionally, heat stress negatively impacts feed conversion, nutrient absorption, and gut health [23,24]. While these findings highlight biological responses, the genetic evaluation of growth trait decline in response to increasing THI levels remains unclear. Specifically, the mechanisms underlying the decline in growth traits with increasing THI remain poorly understood. Additionally, despite the possible influence of pigmentation on heat dissipation, little is known about the genetic correlations between skin color and heat tolerance. These gaps should be addressed to develop effective breeding strategies for improving heat resilience in native chickens.

Therefore, this study aimed to identify the onset of heat stress affecting the growth performance of black-boned and Thai native synthetic chickens and to examine its impact on the genetic potential of these breeds.

## 2. Materials and Methods

This study was reviewed and approved by the Institutional Animal Care and Use Committee of Khon Kaen University on the basis of the ethics of animal experimentation of the National Research Council of Thailand (No. IACUC-KKU-135/64). The study was conducted at the experimental farm of the Network Center for Animal Breeding and Omics Research, Faculty of Agriculture, Khon Kaen University, Thailand.

### 2.1. Animals and Management

**Figure 1:**
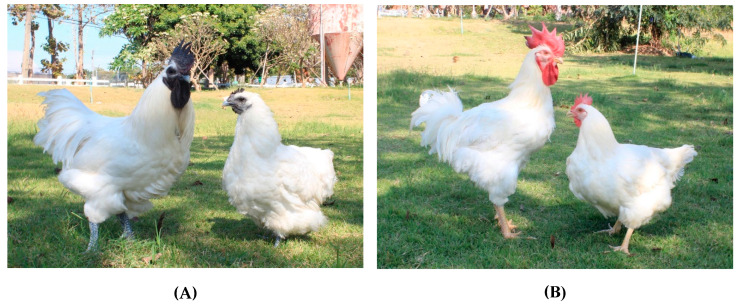
Breed characteristics of (**A**) black-boned and (**B**) Thai native synthetic chickens.

A total of 51,406 records (32,451 male and 18,955 female records) from 4745 black-boned chickens and 32,999 (18,772 male and 14,227 female records) records from 3001 Thai native synthetic chickens (Kaimook e-san), covering body weight (BW), average daily gain (ADG), and absolute growth rate (AGR), were collected across five generations between 2020 and 2024 and utilized in this study. These records represent repeated body weight measurements collected at weekly intervals from hatch to 12 weeks of age. A total of 5425 and 2840 records from black-boned and Thai native synthetic chickens, respectively, were excluded due to missing values or identification as outliers during data screening. Pedigree data were established by tracing all ancestral generations, comprising 6035 black-boned chickens and 4820 Thai native synthetic chickens born between 2019 and 2023. The collected data included BW at hatching and at 4, 8, and 12 weeks of age (BW0, BW4, BW8, and BW12, respectively); ADG at 0–4, 4–8, and 8–12 weeks of age (ADG0–4, ADG4–8, and ADG8–12, respectively); and AGR at hatching and at 4, 8, and 12 weeks of age (AGR0, AGR4, AGR8, and AGR12, respectively). After hatching, each chick was individually weighed, and a leg tag was applied for identification until 4 weeks of age. Thereafter, wing bands were used to maintain consistent individual BW records. All birds were vaccinated against infectious bronchitis, Newcastle disease, and fowl pox in accordance with the standard poultry vaccination schedule. Throughout the experimental period, chickens were provided ad libitum access to clean water and standard commercial broiler feed. The feed was formulated in two phases based on age: from hatching to 4 weeks of age, the diet contained 21% crude protein and 3000 kcal/kg metabolizable energy (ME); from 4 to 12 weeks of age, the grower diet contained 19% crude protein and 2900 kcal/kg ME. This diet was maintained until the conclusion of the experiment at 12 weeks of age. All chickens were reared in open-sided housing with natural ventilation. The lighting program followed a two-phase schedule: from hatching to 4 weeks of age, chicks were provided with continuous light (24 h light: 0 h dark) using 100 W heating lamps; from 4 to 12 weeks of age, birds were exposed to natural daylight. The physical characteristics of the two chicken breeds are illustrated in Figure 1. Black-boned and Thai native synthetic chickens similarly have white plumage. However, black-boned chickens have black skin and shanks, whereas Thai native synthetic chickens have white to yellow skin and shanks. Physical traits such as skin pigmentation, feather coverage, and skin type may influence thermotolerance by affecting heat absorption, heat dissipation, and protection against solar radiation. The black-boned and Thai native synthetic chickens used in this study were developed at Khon Kaen University to improve growth performance, meat quality, and disease resistance in poultry adapted to tropical environments. The Thai native synthetic lines were selectively bred from indigenous genetic resources to preserve local adaptability while enhancing productivity. Black-boned chickens are primarily raised for their high-value meat, culturally prized in Thailand for its perceived medicinal benefits and appeal in niche and health-conscious markets.

Under semi-intensive management, black-boned chickens typically reach a market weight of 1.2–1.4 kg at 12–14 weeks, with a feed conversion ratio (FCR) of 3.0–3.5, survivability exceeding 90% in tropical conditions, and a carcass yield of 68–72%. Similarly, Thai native synthetic chickens reach 1.2–1.6 kg at market age, with comparable FCR (3.0–3.5), survivability (>90%), and slightly higher carcass yield (70–75%).

### 2.2. Air Temperature and Relative Humidity Data

Ambient temperature and relative humidity were continuously recorded inside the pens using three automated sensors data loggers (model: ELITECH GSP-6; accuracy: ±0.5 °C for temperature and ±3% RH for humidity, Elitech Technology, Inc., San Jose, CA, USA). The devices were placed at three locations: one at bird level inside the central housing, one at the front edge of the housing, and one at the rear. Environmental data, including ambient air temperature (Temp) and relative humidity (RH), were measured at 3-h intervals and recorded continuously across all five generations to ensure consistency in THI calculations for each batch of chickens. These data were subsequently used to calculate the THI according to the formula proposed by National Oceanic, & Atmospheric Administration (NOAA) [25]:

THI = (1.8 × Temp + 32) − (0.55 − 0.0055 × RH) × (1.8 × Temp − 26),

where Temp is the average air temperature in degrees Celsius, and RH is the average relative humidity expressed in percentage. The average THI values from day 1 to day 28 were used to assess the effects of heat stress on growth traits for each age group of chickens at weeks 0–4, 4–8, and 8–12, respectively.

### 2.3. Statistical Analysis and Growth Curve Estimation

The BW and ADG data were recorded from the experimental farm, whereas the AGR data were calculated using the Gompertz function [26]. The AGR was also used as another observation value in this study to estimate the genetic parameters for heat stress. The steps of individual AGR analysis are as follows. Step 1: The Gompertz model based on the Marquardt method was performed using the nonlinear (NLIN) procedure of SAS software, version 9.0 (SAS Institute Inc., Cary, NC, USA). Available online: https://www.sas.com (accessed on 10 January 2025) to estimate the growth curve of chicken. The initial parameter values (α,β,γ) were estimated, where α  is the asymptotic live BW (grams), β  the log-function for the proportion of the asymptotic mature weight to be gained after hatching (t = weeks), and γ  is a constant scale that is proportional to the overall growth rate (weeks). This step provided the average growth curve parameters of the population. Step 2: The BW of each chicken was adjusted by fixed effects (sex and hatch), and the residual error was estimated. Thus, the predicted BW ($\hat{y}$) was estimated using overall mean and residual error. Step 3: The initial parameter values from Step 1 were used to estimate individual AGRs. In this step, the values ($\hat{y}$) from Step 2 were used to define the model by taking the function’s first derivative for each parameter (der. a, der. b, and der. c; der = derivative) to analyze the value of parms = a, b, and c. The AGR was calculated based on this equation $\alpha \beta \gamma e^{\left(-\beta e^{-\gamma t}\right)}e^{-\gamma t}$. All data were verified before genetic analysis using the Proc UNIVARIATE procedure with SAS version 9.0 software to examine data distribution, including assessing normality, homogeneity of variance, and data outliers (±3 standard deviation was considered an outlier). Statistical differences were compared by sex using the *Scheffe’s* post-hoc test (*p* < 0.05) in the generalized linear model (GLM procedure) for an unbalanced analysis of variance using the SAS software, version 9.0. The decrease in initial growth characteristics caused by the influence of temperature and RH was analyzed using simple regression analysis.

### 2.4. Estimation of Genetic Parameters

The repeatability test-day model using the reaction norm procedure proposed by Boonkum et al. [10] was employed to estimate the threshold point of heat stress and genetic parameters. Different THI thresholds, from 70 to 80 (THI70 to THI80), were tested in the model. The THI function was created to estimate the decline in BW, ADG, and AGR under heat stress. The repeatability test-day model, THI function, and variance-covariance structure matrix used in this study were defined as follows:

The repeatability test-day model used was as follows:

$$y_{ijkl}={CG}_{i}+{{Age}_{j}+SEX}_{k}[f\left(THI\right)]+a_{0l}+a_{1l}[f\left(THI\right)]\;+p_{0l}+p_{1l}[f\left(THI\right)]+e_{ijkl}$$

where $y_{ijkl}$ is the observation value of BW, ADG, and AGR for chicken l in the contemporary group between hatch and generation (CG) class *i*, age (Age) class *j*, and sex (SEX) class *k*; ${CG}_{i}$ is the fixed effect of the contemporary group (hatch and generation) i; ${Age}_{j}$ is the fixed effect of chicken age *j*; ${SEX}_{k}$ is the fixed effect of chicken sex *k*, which is nested with the THI function ([$f\left(THI\right)$]) to describe the changes in BW, ADG, and AGR in males and females (look at slope of the regression) according to the change in THI values; $a_{0l}$ is the random effect of additive genetic without consideration of heat stress (called “intercept”); $a_{1l}$ is the random effect of additive genetic for heat stress (called “slope”); $p_{0l}$ is the random effect of permanent environmental without consideration of heat stress; $p_{1l}$ is the random effect of permanent environmental of heat stress; $e_{ijkl}$ is the random effect of residual; and $f\left(THI\right)$ is a function of the THI.

The following THI function was used:

$$f\left(THI\right)=\left\{\begin{array}{c} \;\;\;\;\;\;\;0,\;\;THI\leq {THI}_{threshold}\;(no\;heat\;stress)\\ THI-{THI}_{threshold},\;\;THI>{THI}_{threshold}\;(heat\;stress) \end{array}\right.$$

The THIs included in the repeatability test-day model were set at various critical values or threshold points. Different THI thresholds, from 70 to 80 (THI70 to THI80), were tested in the model. The final THI thresholds (best-fit model) were determined based on the lowest values of minus twice the logarithm of the likelihood (−2logL) and Akaike’s information criterion (AIC) and were biologically validated through observed inflection points in growth–trait slopes.

The variance-covariance structure used was as follows:

$$Var\left[\begin{array}{c} a_{0}\\ a_{1}\\ p_{0}\\ p_{1}\\ e \end{array}\right]=\left[\begin{array}{ccccc} A\sigma_{a0}^{2} & A\sigma_{a01} & 0 & 0 & 0\\ A\sigma_{01} & A\sigma_{a1}^{2} & 0 & 0 & 0\\ 0 & 0 & I\sigma_{p0}^{2} & I\sigma_{p01} & 0\\ 0 & 0 & I\sigma_{p01} & I\sigma_{p1}^{2} & 0\\ 0 & 0 & 0 & 0 & I\sigma_{e}^{2} \end{array}\right]$$

where $\sigma_{a0}^{2}$ is additive genetic variance without consideration of heat stress; $\sigma_{a1}^{2}$ is additive genetic variance for heat stress; $\sigma_{p0}^{2}$ is the permanent environmental variance without consideration of heat stress; $\sigma_{p1}^{2}$ is the permanent environmental variance of heat stress;${\;\sigma }_{e}^{2}$ is the residual variance; A is the numerator relationship matrix; and I is an identity matrix. Variance components were estimated with the average information-restricted maximum likelihood algorithm (AIREMLF90 module) from the BLUPF90+ software package based on pedigree data [27]. The heritability values of the BW, ADG, and AGR under hot–humid climates were estimated by Ravagnolo and Misztal [28] with the following equation:

$$h^{2}=\frac{\sigma_{a0}^{2}+\sigma_{a1}^{2}+{2\sigma }_{a01}}{\sigma_{a0}^{2}+\sigma_{a1}^{2}+{2\sigma }_{a01}+\sigma_{p0}^{2}+\sigma_{p1}^{2}+{2\sigma }_{p01}{+\sigma }_{e}^{2}}$$

Genetic correlations $\left(r_{g}\right)$ between the intercept and slope were calculated. These values refer to the genetic components of two or more traits. It measures the extent to which the same genes or sets of genes influence multiple traits simultaneously. The correlations between the intercept and slope of the permanent environmental effects $\left(r_{p}\right)$ were calculated as follows:

$$r_{g}=\frac{{COV\sigma }_{a0,a1}}{\sqrt{\sigma_{a0}^{2}*\sigma_{a1}^{2}}}\;{,r}_{p}=\frac{{COV\sigma }_{p0,p1}}{\sqrt{\sigma_{p0}^{2}*\sigma_{p1}^{2}}}$$

## 3. Results

### 3.1. Determination of the Onset of Heat Stress Threshold Using THI

The onset of heat stress in the black-boned and Thai native synthetic chickens was evaluated at THI70–THI80 (Table 1). The most appropriate THI threshold was determined in accordance with two statistical criteria: −2logL and AIC. Lower values of these criteria indicate better model fit and, consequently, more accurate thresholds for defining the environmental stress point at which heat begins to negatively impact physiological or productive traits. For the black-boned chickens, the best model fit was observed at THI72, which had the lowest −2logL and AIC values (both set to zero). Thus, THI72 is the optimal threshold for detecting the onset of heat stress in this breed. At this point, any THI value greater than 72 is likely to result in measurable heat stress effects on BW, ADG, or BrC. By contrast, for the Thai native synthetic chickens, the optimal THI threshold was determined to be THI76, at which −2logL and AIC reached their minimum values (0).

### 3.2. Growth Comparisons

**Figure 2:**
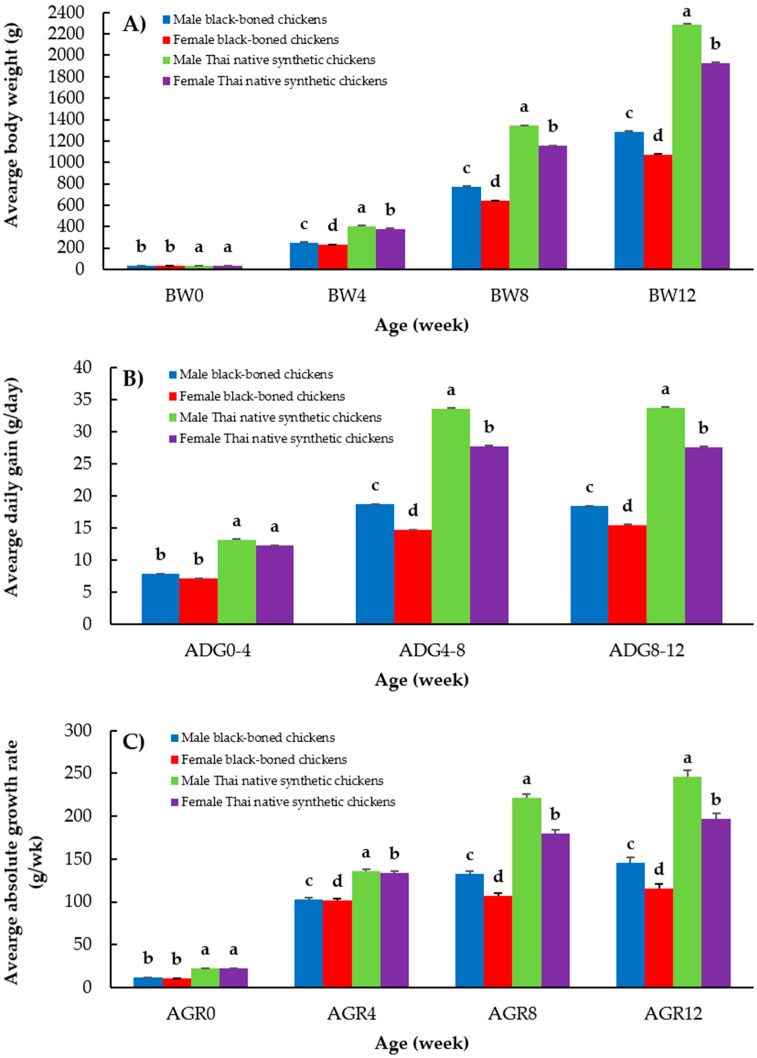
Least-squares means of (**A**) body weight (BW), (**B**) average daily gain (ADG), and (**C**) absolute growth rate (AGR) traits separated by sex and chicken breeds in black-boned and Thai native synthetic chickens; a, b, c, and d: Means for the trait with different letters differ significantly at *p* < 0.05. BW0, BW4, BW8, and BW12: hatching weight, body weight at 4, 8, and 12 weeks of age, respectively; ADG0–4, ADG4–8, and ADG8–12: average daily gain during hatching to 4, 4 to 8, and 8 to 12 weeks of age, respectively; AGR0, AGR4, AGR8, and AGR12: absolute growth rate at hatching, 4, 8, and 12 weeks of age, respectively.

Least-squares means for BW, ADG, and AGR, stratified by sex and chicken breed, in the black-boned and Thai native synthetic chickens are presented in Figure 2. As shown in Figure 2A, the initial body weight (BW0) was slightly higher in the Thai native synthetic chickens (male: 37.34 ± 0.10 g; female: 37.15 ± 0.10 g) than in the black-boned chickens (male: 33.25 ± 0.08 g; female: 32.95 ± 0.08 g) (*p* < 0.05). This difference in weight characteristics increased with age. By 12 weeks of age (BW12), male Thai native synthetic chickens achieved a mean BW of 2287.28 ± 7.14 g, whereas females reached 1927.91 ± 7.12 g. By contrast, male and female black-boned chickens exhibited significantly lower BW12 values of 1287.97 ± 5.63 g and 1071.97 ± 5.38 g, respectively (*p* < 0.05). As illustrated in Figure 2B, the Thai native synthetic chickens demonstrated consistently higher ADG across all age intervals than their black-boned counterparts (*p* < 0.05). The most notable differences occurred during the 4–8 and 8–12 week periods. In specific, male Thai native synthetic chickens had ADG values of 33.51 ± 0.12 g/day (4–8 weeks) and 33.71 ± 0.13 g/day (8–12 weeks), whereas male black-boned chickens had ADG values of 18.66 ± 0.10 g/day (4–8 weeks) and 18.02 ± 0.11 g/day (8–12 weeks). A similar trend was observed in females, although at slightly lower magnitudes. The AGR data in Figure 2C support the superior growth performance of the Thai native synthetic chickens over the black-boned chickens. At 12 weeks (AGR12), the AGR values in male and female Thai native synthetic chickens were 213.3 ± 1.77 g/week and 138.9 ± 1.76 g/week, respectively, which were significantly higher than the corresponding values in the black-boned chickens (males: 122.1 ± 1.39 g/week; females: 73.2 ± 1.33 g/week) (*p* < 0.05). Notably, the black-boned chickens exhibited relatively stable AGR at 4–12 weeks of age, whereas the Thai native synthetic chickens, particularly males, showed steeper growth trajectories during the same period.

**Figure 3:**
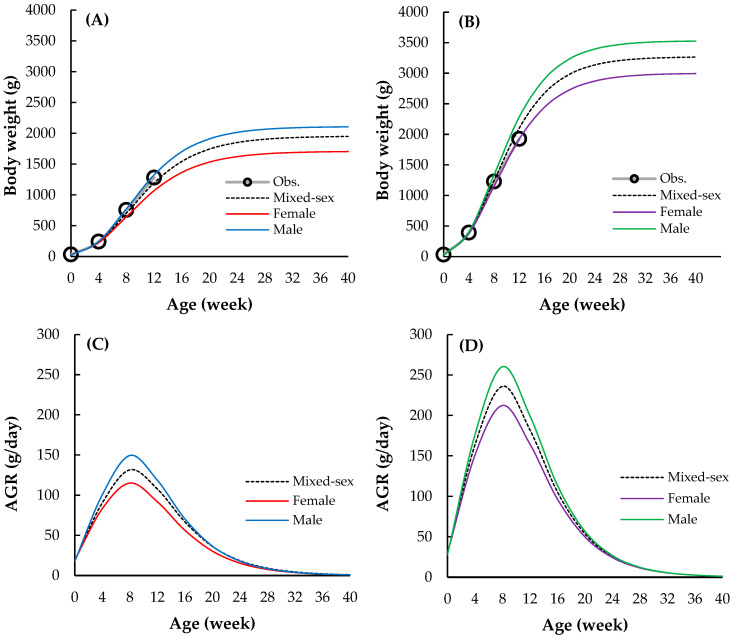
Growth patterns of (**A**) body weight in black-boned (32,451 male and 18,955 female records), and (**B**) Thai native synthetic chickens (18,772 male and 14,227 female records), along with the absolute growth rate (AGR) estimated using the Gompertz function in (**C**) black-boned and (**D**) Thai native synthetic chickens. Obs. means observed.

The BW and AGR trajectories differed markedly between the black-boned and Thai native synthetic chickens across all age stages, as presented in Figure 3. At hatching (0 weeks), both breeds exhibited similar BWs (black-boned: 33.5 g; Thai native synthetic: 32.2 g) (Figure 3A,B). However, as age progressed, divergence in growth performance became increasingly evident. At 12 weeks of age, the Thai native synthetic chickens attained a substantially higher average BW (2107.24 g) than the black-boned chickens (1195.31 g). By 40 weeks, the Thai native synthetic chickens reached a final BW of 3264.03 g, with males and females weighing 3526.70 g and 2995.45 g, respectively. By contrast, the black-boned chickens reached a lower terminal BW of 1948.54 g, with males at 2105.23 g and females at 1703.40 g. Sexual dimorphism was consistently observed in both breeds. Males outperformed females in BW at all age points, with the sex-related disparity becoming more pronounced after 8 weeks. Notably, male Thai native synthetic chickens exhibited a rapid increase in BW between 8 and 20 weeks, whereas male black-boned chickens followed a more gradual growth trajectory. Figure 3C,D illustrate the AGR profiles across ages, which highlighted distinct genetic growth potentials between the two breeds. At hatching, the Thai native synthetic chickens exhibited a higher AGR (28.81 g/week) than the black-boned chickens (18.84 g/week), with this gap widening during the early growth stages. The peak AGR occurred at 8 weeks in both breeds, with 260.27 g/week and 212.34 g/week in male and female Thai native synthetic chickens, respectively, and 149.40 g/week and 115.21 g/week in male and female black-boned chickens, respectively. Between 8 and 20 weeks, the Thai native synthetic chickens consistently demonstrated higher growth rates than the black-boned chickens. AGR declined in both lines after 12 weeks, reflecting the expected physiological deceleration as the birds approached maturity. However, this decline was more pronounced in the black-boned chickens, whose AGR dropped to 1.04 g/week by 40 weeks, compared to 1.15 g/week in the Thai native synthetic chickens.

### 3.3. Heritability Estimates

**Figure 4:**
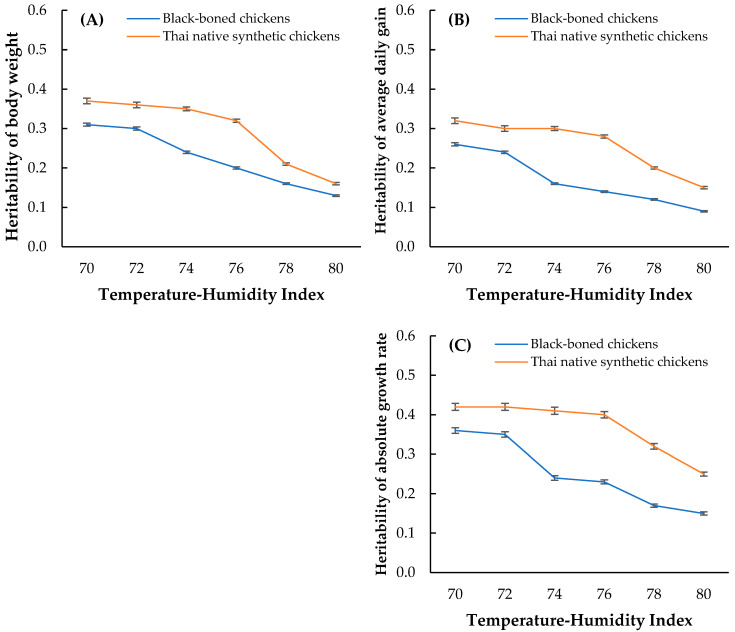
Estimated heritability of (**A**) body weight, (**B**) average daily gain, and (**C**) absolute growth rate in black-boned chickens (orange line) and Thai native synthetic chickens (blue line).

The estimated heritability values for BW, ADG, and AGR in the black-boned and Thai native synthetic chickens are presented in Figure 4. At THI70–THI80, BW heritability declined from 0.31 to 0.13 in the black-boned chickens and from 0.37 to 0.16 in the Thai native synthetic chickens. Although both breeds exhibited sensitivity to increasing heat stress, the synthetic line consistently maintained higher BW heritability at all THI levels. The average BW heritability across the heat stress gradient was 0.21 in the black-boned chickens and 0.28 in the Thai native synthetic chickens. A similar declining trend was observed for ADG heritability, with values decreasing from 0.26 to 0.09 in the black-boned chickens and from 0.32 to 0.15 in the Thai native synthetic chickens as the THI was increased. On average, ADG heritability was 0.15 in the black-boned chickens and 0.25 in the Thai native synthetic chickens. Among all traits, AGR exhibited the highest heritability at the baseline THI (70), with estimates of 0.42 in the Thai native synthetic chickens and 0.36 in the black-boned chickens. Although heritability declined at THI80 (to 0.25 and 0.15, respectively), the Thai native synthetic chickens retained a superior overall genetic potential for growth under heat stress, as reflected by their higher average AGR heritability than the black-boned chickens (0.36 vs. 0.23).

### 3.4. Genetic and Phenotypic Correlation Estimates

The genetic and phenotypic correlations between growth traits and THI values at THI72 and THI76 for the black-boned and Thai native synthetic chickens are presented in Table 2. Across both breeds, strong negative correlations were observed. In the black-boned chickens, the genetic correlations between THI and BW, ADG, and AGR were −0.69, −0.74, and −0.79, respectively, at THI72. These values became more pronounced at THI76, with correlations of −0.77 for BW, −0.85 for ADG, and −0.89 for AGR. Corresponding phenotypic correlations also reflected this trend, ranging from −0.77 to −0.95, highlighting a strong environmental influence on performance reduction under higher heat stress. The Thai native synthetic chickens exhibited weaker but still substantial negative correlations. At THI72, genetic correlations between THI and BW, ADG, and AGR were −0.50, −0.59, and −0.68, respectively. These correlations intensified at THI76 (−0.62, −0.70, and −0.82, respectively). Phenotypic correlations similarly followed a declining trend with increasing THI, ranging from −0.69 to −0.89.

### 3.5. Trait Reductions Under Heat Stress

**Figure 5:**
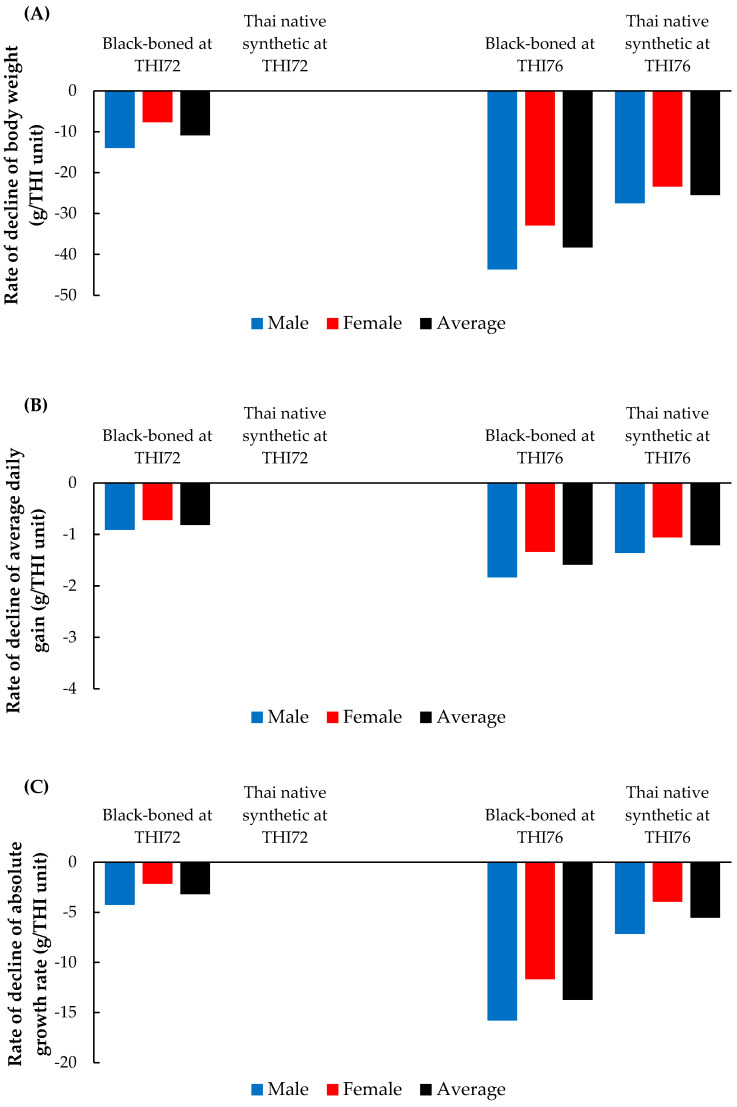
Rate of decline in (**A**) body weight, (**B**) average daily gain, and (**C**) absolute growth rate in black-boned and Thai native synthetic chickens at THI72 and THI76.

The effects of heat stress on BW, ADG, and AGR were evaluated at THI72 and THI76 in the black-boned and Thai native synthetic chickens (Figure 5). At THI72, the black-boned chickens exhibited marked reductions in BW, with an average loss of −10.9 g per THI unit. Male chickens showed a more pronounced decline (−14.0 g/THI unit) than females (−7.7 g/THI unit). By contrast, the Thai native synthetic chickens demonstrated negligible changes in BW (−0.01 g/THI unit for both sexes). Similar patterns were observed in ADG and AGR, wherein the black-boned chickens experienced average reductions of −0.87 g/day/THI unit and −3.20 g/week/THI unit, respectively. Conversely, the Thai native synthetic chickens maintained relative stability, with minimal decreases (−0.01 g/THI unit for both traits), suggesting greater thermal tolerance under mild heat stress. At THI76, the black-boned chickens experienced further deterioration in growth traits. BW declined by an average of −25.5 g/THI unit, with males exhibiting losses as high as −38.3 g/THI unit. ADG and AGR decreased more substantially, reaching −1.74 g/day/THI unit and −12.24 g/week/THI unit, respectively. Under the same THI level, the Thai native synthetic chickens also exhibited BW reductions averaging −25.5 g/THI unit, despite previously demonstrating stability at THI72. ADG and AGR similarly declined (−1.26 g/day/THI unit and −5.55 g/week/THI unit, respectively), particularly among females.

### 3.6. EBV Trends Across THI Level

**Figure 6:**
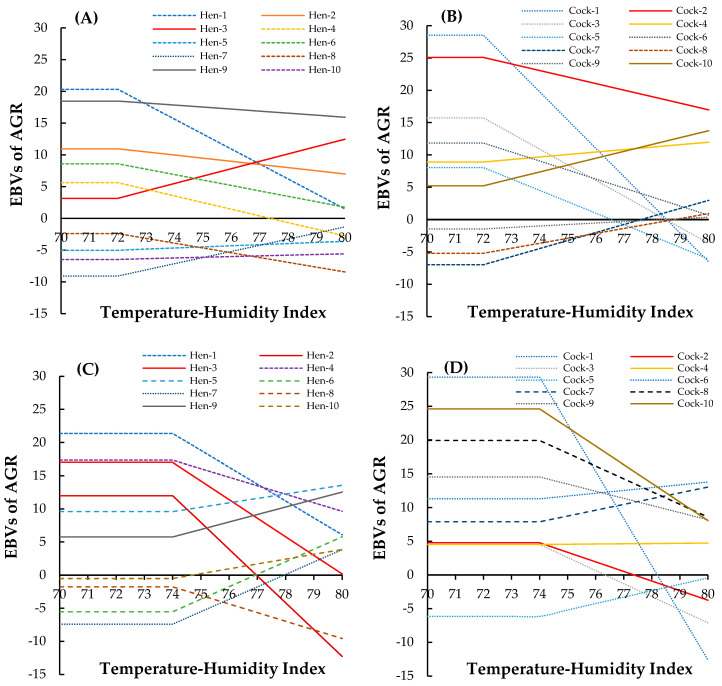
Integrated reaction norm-based on estimated breeding values (EBVs) for AGR under increasing THI conditions in (**A**) 10 randomly selected hens, (**B**) 10 randomly selected cocks in black-boned chickens, (**C**) 10 randomly selected hens, and (**D**) 10 randomly selected cocks in Thai native synthetic chickens.

The estimated breeding values (EBVs) of randomly selected individuals from both sexes and breeds for AGR at THI70–THI80 are presented in Figure 6. As shown in Figure 6A,B, both sexes exhibited steep declines in EBVs for AGR as the THI increased. Although individual variation was observed, most birds showed a linear or near-linear decrease in genetic potential for growth under heat stress. At THI80, several individuals, particularly cocks, displayed negative EBVs. Interestingly, hens generally maintained slightly higher EBVs than cocks under the same conditions. In contrast to the black-boned chickens, the Thai native synthetic chickens (Figure 6C,D) demonstrated greater variability in response to rising THI. Some birds, both cocks and hens, retained positive or relatively stable EBVs even as the THI approached 80.

## 4. Discussion

Heat stress represents a critical constraint to poultry production, particularly in tropical regions, where elevated ambient temperatures and high humidity significantly impair growth performance. Consequently, the development of native chicken genetic lines with enhanced thermotolerance but without compromising productive traits is essential for sustainable production. 

This study provides novel insights into the genetic responses of black-boned and Thai native synthetic chickens to increasing thermal stress, as indicated by the temperature–humidity index. By assessing the onset of heat stress and its effects on growth traits, it offers a comparative model for selecting heat-resilient, productive native chicken lines for tropical environments. The optimal THI thresholds—THI72 for black-boned and THI76 for Thai native synthetic chickens—highlight breed-specific differences in thermotolerance. The lower threshold in black-boned chickens suggests greater susceptibility to heat stress, with performance declines in BW, ADG, and BrC. This may be partly due to their dark-pigmented skin absorbing more heat, reducing heat dissipation efficiency compared to the more heat-adapted native breed [29,30,31]. Melanin, a key pigment abundantly present in black-boned chickens, has been shown to influence heat absorption and retention due to its high thermal conductivity and absorptivity [32,33]. Darker plumage and skin pigmentation can enhance solar heat gain, which may be either advantageous or detrimental depending on the environmental context [34]. In black-boned chickens, which exhibit extensive hyperpigmentation due to fibromelanosis, such pigmentation may increase susceptibility to thermal stress under high ambient temperatures by facilitating excessive heat absorption [35]. While melanin confers protection against ultraviolet radiation and may exert antioxidative effects [36], its thermogenic properties can lead to elevated core body temperatures under heat stress, thereby negatively affecting growth performance and survivability in hot climates. This dual role highlights the importance of considering pigmentation as a thermophysiological trait in poultry breeding and climate-adaptation strategies. In contrast, Thai native synthetic chickens maintained stable performance up to THI76, indicating superior thermal resilience, likely due to hybrid vigor and the use of heat-adapted parental lines [11,12]. Similar trends have been observed in commercial × native crossbreds, where heterosis enhances both heat tolerance and growth potential [37,38,39].

Growth performance data underscore the genetic differences between the two breeds. From hatching to 12 weeks, Thai native synthetic chickens consistently outperformed black-boned chickens in BW, ADG, and AGR, with the disparity becoming more pronounced with age—particularly in males. This trend reflects not only selection for growth efficiency but also an enhanced capacity to sustain metabolic function under suboptimal conditions. These findings support the hypothesis that incorporating environmental robustness into breeding objectives can indirectly enhance heat tolerance [10,14]. However, the magnitude of sex-based differences was greater in the Thai native synthetic line, especially after 8 weeks, suggesting differential expression of growth-regulating genes and heightened endocrine sensitivity in males under both favorable and stressful environments. Previous studies indicated that domestication and selection have led to distinct physiological and behavioral adaptations in males, including enhanced endocrine responsiveness and altered gene expression profiles [40,41]. AGR peaked at 8 weeks, consistent with typical growth patterns in native and dual-purpose chickens. Although growth declined thereafter, Thai native synthetic chickens maintained higher post-peak AGR than black-boned chickens, suggesting a sustained growth advantage into maturity. This trait may offer practical benefits for dual-purpose production systems where longer growing periods are economically feasible. Thai native synthetic chickens exhibit superior growth performance and heat resilience compared to black-boned chickens, supporting their potential as a genetic resource for improving local poultry production under climate variability. Furthermore, the identification of breed-specific temperature–humidity index thresholds provide actionable metrics for environmental management, breeding strategies, and heat stress mitigation in native chicken systems.

The decline in heritability estimates with increasing THI in both genetic groups aligns with previous reports indicating that environmental stressors reduce the expression of additive genetic variance in poultry and other livestock species [10,13,42]. For example, BW heritability decreased from 0.31 to 0.13 in black-boned chickens and from 0.37 to 0.16 in Thai native synthetic chickens as THI increased from 70 to 80. These results support the findings of Al-Abdullatif and Azzam [43], who reported that high ambient temperatures impair feed intake and metabolic efficiency, thereby elevating environmental variance in growth traits. Notably, Thai native synthetic chickens consistently exhibited higher heritability estimates across all THI levels—averaging 0.28 (BW), 0.25 (ADG), and 0.36 (AGR)—suggesting a greater genetic capacity to sustain growth under heat stress. This may be attributed to adaptive alleles inherited from heat-tolerant ancestral lines. Their stable trait expression at moderate THI (e.g., minimal change in BW, ADG, and AGR at THI 72) further supports the hypothesis of genetic buffering under thermal stress.

The negative genetic and phenotypic correlations between THI and growth traits in black-boned and Thai native synthetic chickens clearly indicate the detrimental effects of heat stress on performance. As THI increased from 72 to 76, reductions in BW, ADG, and AGR were more pronounced, particularly in black-boned chickens. These results align with previous findings that elevated ambient temperatures impair feed intake, metabolic efficiency, and endocrine function, thereby suppressing growth [13,20]. In black-boned chickens, strong genetic correlations at THI72 and THI76 (up to −0.89 for AGR) suggest a limited genetic capacity to cope with heat stress. This observation supports prior reports that indigenous breeds, although adapted to low-input systems, may lack specific genetic mechanisms for resilience under acute thermal stress [44,45]. Similarly, the high magnitude of phenotypic correlations (up to −0.95) indicates that environmental effects—likely due to reduced thermoregulatory efficiency and increased oxidative stress—are key drivers of performance decline under heat stress [15,21]. In contrast, Thai native synthetic chickens exhibited weaker correlations between THI and growth traits, especially at THI72, suggesting a stronger genetic foundation for growth under moderate heat loads. This resilience may result from heterosis or prior selection for thermotolerant traits in the parental lines [11]. Even at THI76, genetic correlations remained less severe than in black-boned chickens, implying the presence of alleles that confer partial heat tolerance. These findings are consistent with genomic studies showing that crossbred or synthetically improved native chickens often demonstrate enhanced thermotolerance due to favorable gene interactions [37,46]. The divergent correlation patterns between the two genetic groups highlight the greater genetic and phenotypic robustness of Thai native synthetic chickens, making them promising candidates for the development of climate-resilient poultry lines. Future research should apply genomic selection and functional gene mapping to identify loci associated with heat tolerance and improve breeding strategies.

Despite the resilience of Thai native synthetic chickens at THI 72, a performance reversal occurred at THI 76, where they exhibited greater absolute reductions in BW (−68.3 g), ADG (−1.74 g/day), and AGR (−12.24 g/week) compared to black-boned chickens. This sharp decline suggests that Thai native synthetic chickens may reach a critical physiological threshold beyond which homeostatic mechanisms fail. These observations are consistent with previous reports indicating nonlinear responses to heat stress in poultry, particularly when THI exceeds tolerance limits. Elevated THI adversely affects physiological functions, leading to reduced productivity and impaired welfare, as evidenced by altered metabolic and behavioral patterns that compromise health and reproductive capacity [16,19,47]. Furthermore, female synthetic chickens appeared more susceptible at high THI, possibly due to distinct thermoregulatory responses or hormonally mediated metabolic adjustments [48]. In contrast, black-boned chickens exhibited gradual declines across THI levels. Although their lower heritability estimates and consistent trait deterioration suggest limited genetic potential for heat tolerance improvement, their moderate reductions at THI 76 imply the presence of alternative physiological or behavioral coping strategies, such as decreased activity or efficient evaporative cooling [49]. The low standard errors of heritability estimates (0.003–0.007) support the robustness and reliability of variance component estimation under heat stress. Overall, these findings highlight the genetic potential of Thai native synthetic chickens for selection programs targeting heat resilience and growth performance.

The results of this study demonstrate significant genetic variation in the response of black-boned and Thai native synthetic chickens to escalating heat stress, as assessed by estimated breeding values (EBVs) for average growth rate (AGR) across temperature–humidity index gradients. A consistent decline in EBVs—particularly among hens—highlights the adverse effects of heat stress on genetic growth potential. This pattern aligns with previous findings showing that poultry growth performance, especially in tropical environments, is sensitive to elevated temperatures due to reduced feed intake, hormonal disruption, and metabolic inefficiencies [13,15,18]. The linear or near-linear decrease in AGR EBVs, especially in black-boned chickens, underscores this line’s genetic vulnerability to heat stress. These findings are consistent with prior reports suggesting that native or minimally selected populations lack the genetic buffering capacity against environmental challenges compared to more intensively improved lines [50,51]. The consistently lower EBVs in hens across both genetic lines also indicate potential sex-specific sensitivity to thermal stress, likely linked to differences in metabolic rate, endocrine profiles, or reproductive investment. Conversely, the wider range of AGR EBVs in the synthetic line, particularly among cocks, indicates the presence of heat-tolerant genotypes. Notably, several individuals maintained flat or slightly positive EBV trajectories at high THI levels (THI 78–80), suggesting the existence of alleles conferring thermal resilience. Similar trends have been observed in studies identifying candidate genes or quantitative trait loci associated with heat tolerance and growth stability in poultry [37,52]. This genotypic resilience is critical for breeding programs aimed at enhancing climate adaptation. Furthermore, the intra-line variability observed within the synthetic line underscores the importance of targeted selection for heat tolerance. Identifying genetically stable individuals under thermal stress provides a valuable foundation for marker-assisted or genomic selection to improve resilience without compromising growth. These strategies are increasingly vital given the projected intensification of heat waves due to climate change [53].

## 5. Conclusions

The optimal THI thresholds for detecting heat stress were THI72 for black-boned chickens and THI76 for Thai native synthetic chickens based on model fit (−2logL and AIC). The synthetic chickens consistently outperformed the black-boned chickens in BW, ADG, and AGR, with higher values across all age points. Heritability estimates for BW, ADG, and AGR declined with rising THI in both breeds, but the Thai native synthetic chickens maintained higher genetic potential under stress. Trait reductions under heat stress were more severe in the black-boned chickens at THI72, whereas the Thai native synthetic chickens showed resilience until THI76, after which their performance dropped. EBV analyses revealed greater thermal resilience and genetic variability in the Thai native synthetic chickens, particularly among males, than in the black-boned chickens. Overall, Thai native synthetic chickens exhibited superior growth performance and heat stress tolerance over black-boned chickens, highlighting their suitability for breeding programs in hot climates. Therefore, chicken breeding programs should be considered to enhance genetic production potential and adapt to harsh climatic conditions.

## Figures and Tables

**Table 1:** Onset of heat stress in black-boned and Thai native synthetic chickens.

Chicken Breed	Black-Boned Chicken		Thai Native Synthetic Chicken	
Temperature-Humidity Index (THI)/Statistic Criteria	−2logL	AIC	−2logL	AIC
THI70	+59	+59	+240	+240
THI71	+22	+22	+175	+175
THI72	0	0	+153	+153
THI73	+48	+48	+72	+72
THI74	+82	+82	+59	+59
THI75	+95	+95	+22	+22
THI76	+129	+129	0	0
THI77	+225	+225	+48	+48
THI78	+428	+428	+135	+135
THI79	+439	+439	+268	+268
THI80	+462	+439	+392	+392

−2logL = minus twice the logarithm of the likelihood; AIC = Akaike’s information criterion.

**Table 2:** Genetic and phenotypic correlations between growth traits and varying temperature–humidity index (THI) levels in black-boned and Thai native synthetic chickens.

Parameters	Genetic Correlations		Phenotypic Correlations	
Chicken Breeds and Traits	THI72	THI76	THI72	THI76
Black-boned chickens				
BW	−0.69	−0.77	−0.77	−0.85
ADG	−0.74	−0.85	−0.86	−0.92
AGR	−0.79	−0.89	−0.89	−0.95
Thai native synthetic chickens				
BW	−0.50	−0.62	−0.69	−0.77
ADG	−0.59	−0.70	−0.72	−0.78
AGR	−0.68	−0.82	−0.79	−0.89

## Data Availability

The original contributions presented in the study are included in the article, further inquiries can be directed to the corresponding author.
